# Impacts of biofilms on the conversion of cellulose

**DOI:** 10.1007/s00253-020-10595-y

**Published:** 2020-04-26

**Authors:** Simone Brethauer, Robert L. Shahab, Michael H. Studer

**Affiliations:** School of Agricultural, Forest and Food Sciences, Laboratory of Biofuels and Biochemicals, Bern University of Applied Sciences (BFH), 3052 Zollikofen, Switzerland

**Keywords:** Biofilm, Cellulose degradation, Cellulolytic enzymes, Solid state fermentation, Microbial communities

## Abstract

**Abstract:**

Lignocellulose is a widely available renewable carbon source and a promising feedstock for the production of various chemicals in biorefineries. However, its recalcitrant nature is a major hurdle that must be overcome to enable economic conversion processes. Deconstruction of lignocellulose is part of the global carbon cycle, and efficient microbial degradation systems have evolved that might serve as models to improve commercial conversion processes. Biofilms—matrix encased, spatially organized clusters of microbial cells and the predominating lifestyle in nature—have been recognized for their essential role in the degradation of cellulose in nature, e.g., in soils or in the digestive tracts of ruminant animals. Cellulolytic biofilms allow for a high concentration of enzymes at the boundary layer between the solid substrate and the liquid phase and the more complete capture of hydrolysis products directly at the hydrolysis site, which is energetically favorable. Furthermore, enhanced expression of genes for carbohydrate active enzymes as a response to the attachment on solid substrate has been demonstrated for cellulolytic aerobic fungi and anerobic bacteria. In natural multispecies biofilms, the vicinity of different microbial species allows the creation of efficient food webs and synergistic interactions thereby, e.g., avoiding the accumulation of inhibiting metabolites. In this review, these topics are discussed and attempts to realize the benefits of biofilms in targeted applications such as the consolidated bioprocessing of lignocellulose are highlighted.

**Key Points:**

- *Multispecies biofilms enable efficient lignocellulose destruction in the biosphere.*
- *Cellulose degradation by anaerobic bacteria often occurs by monolayered biofilms.*
- *Fungal biofilms immobilize enzymes and substrates in an external digestion system.*
- *Surface attached cultures typically show higher expression of cellulolytic enzymes.*

## Introduction

**Fig. 1:**
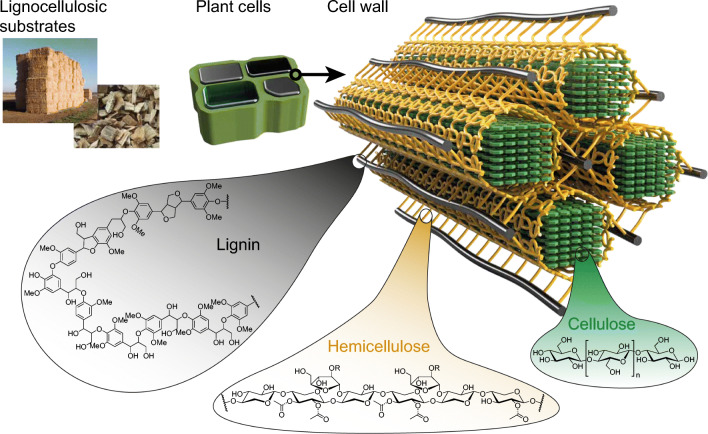
Components and structure of lignocellulosic plant cell walls. Lignocellulosic plant cell walls are mainly composed of cellulose, hemicellulose, and lignins. Cellulose is a homopolysaccharide of d-glucose monomers which are glycosidically linked in the β-(1–4) configuration. The repetitive unit is cellobiose. Multiple linear cellulose chains form an elementary fibril stabilized by hydrogen bonds. Multiple bundles of cellulose fibers coagulate and form macrofibrils. Hemicellulose is in contrast to cellulose an often branched heteropolysaccharide composed of glucose and dependent of the plant species different pentoses such as xylose, mannose, and arabinose. The monomeric building blocks of lignin are coniferyl alcohol, ρ-coumaryl alcohol and sinapyl alcohol which are linked by carbon-carbon and ether linkages. The structure of lignin is adapted from Rozmysłowicz et al. (2019). The three polymers cellulose, hemicellulose and lignin form the highly recalcitrant composite structure lignocellulose. Please note that the 3D structure of the composite material is simplified for better visualization. As example, the number of elementary cellulose fibers which congregate to micro- and macrofibrils is significantly higher

Global climate change leads to far-reaching environmental and social impacts and drives the pursuit of a transition towards a low carbon economy which represents not only a significant opportunity but also an enormous challenge. Lignocellulose—as the largest renewable source of fixed carbon—has attracted considerable attention as an alternative feedstock to petroleum. However, its recalcitrant nature is a major hurdle to microbial degradation and limits its economic use in industrial conversions to fuels and chemicals (Himmel et al. 2007). Lignocellulose is an intimate complex of the polysaccharides cellulose and hemicellulose and the phenolic macromolecule lignin (Fig. 1). Fungi and bacteria express a diverse set of hydrolytic and accessory enzymes that function synergistically and have evolved different strategies to depolymerize plant biomass (see Box 1). In natural ecosystems, these enzymes often are produced by and perform their tasks in conjunction with biofilms (see box 2), which is the prevailing lifestyle of most microorganisms (Flemming and Wuertz 2019; Sivadon et al. 2019).

This review provides an overview of the impact of biofilms on cellulose degradation in aerobic and anaerobic ecosystems such as soils or the ruminant digestive tract as well as in defined laboratory systems. Furthermore, attempts to realize the benefits of biofilms in targeted applications such as the production of cellulolytic enzymes and the direct fermentation of cellulose to different target products are highlighted.

### The role of biofilms in lignocellulose degradation in the biosphere

Approximately 85% of the decomposition of organic material is caused by natural microbial communities comprised of fungi, bacteria, algae, archaea, and protozoa (Bärlocher 2016; Burmølle et al. 2012). In nature, lignocellulose is degraded under both aerobic and anaerobic conditions in various ecosystems, whereas profound differences exist in, e.g., the cellulolytic enzyme systems, the cell mass yield, and the final end products (Wei et al. 2009).

### Lignocellulose degradation in predominately aerobic ecosystems

Under aerobic conditions, non-complexed extracellular cellulases are secreted (see Box 1) and typically high cell masses are produced with CO_2_ and H_2_O as the accompanying metabolic end products of the respiratory chain. Ecosystems, where aerobic cellulolytic microbial communities dominate, are for example streams and soils (Wei et al. 2009).

**Fig. 2:**
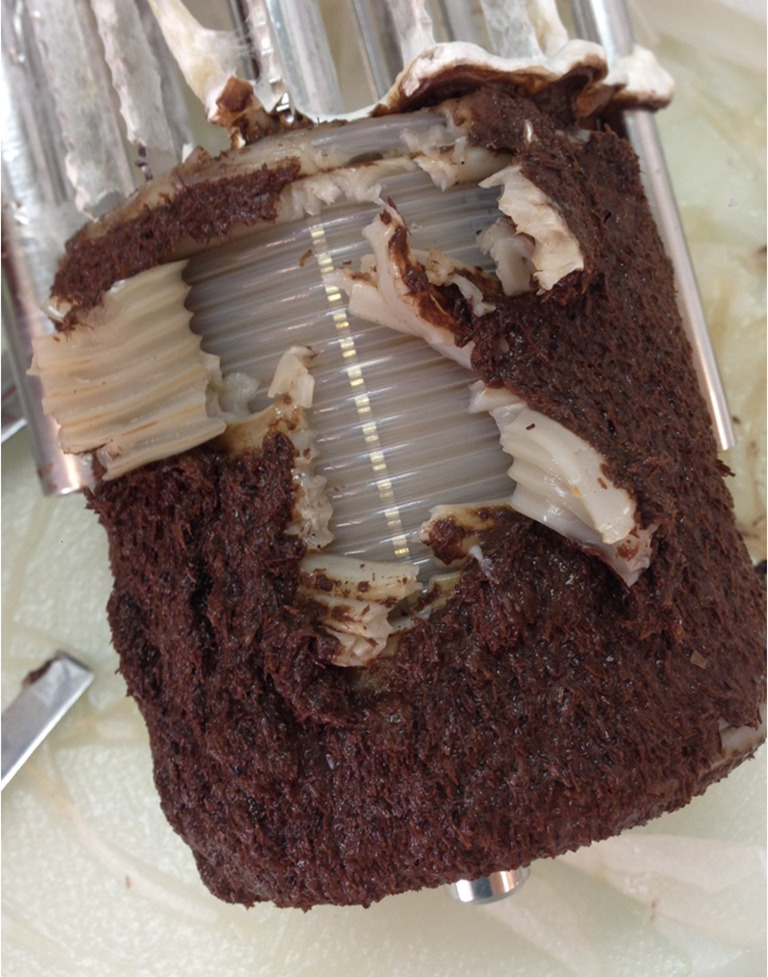
Picture of an *Irpex lacteus* biofilm that has sequestered solid beechwood particles from the liquid phase (Brethauer et al. 2017)

In streams and rivers, biofilms consisting of prokaryotic and eukaryotic microorganisms are formed on practically every available surface, visible by the typical slimy appearance (Bärlocher 2016). Organic detritus such as leaves, needles, and twigs derived from the riparian vegetation is the dominating carbon source and also serves as a substrate for biofilm formation. The amount of microbial biomass and the community structure varies with the type of substrate, but fungal diversity is typically higher than bacterial diversity (Hellal et al. 2016; Gollady and Sinsabaugh 1991). The biofilm matrix consisting of EPS allows for the retention of extracellular enzymes mainly by their interaction with polysaccharides. Many of these enzymes are involved in the degradation of soluble as well as solid biopolymers including cellulose or organic particles in general. For example, endocellulases and β-glucosidases have been found in river biofilms. The matrix also sequesters dissolved and particulate nutrients from the surrounding water phase as can be observed also in laboratory systems (Fig. 2). Overall, an external digestive system is generated, that minimizes the loss of enzymes and corresponding depolymerization products to the flowing water phase (Flemming and Wingender 2010).

Soils differ in many aspects from freshwater ecosystems (Bärlocher 2016). Soil is the most heterogenous component of the biosphere and humidity and temperature fluctuate on a short time scale (Flemming and Wuertz 2019). The soil microbiome represents the most biologically diverse community on land and is essential in driving biogeochemical cycles (Crowther et al. 2019). Dead organic matter such as wood and leaves represent the major solid organic carbon source, while plant roots also exude soluble compounds. Cellulolytic and ligninolytic aerobic fungi (often *Ascomycota* and *Basidiomycota*) and bacteria (e.g., *Streptomyces, Micromonospora, Bacillus, Cellulomonas* and *Cytophaga*) interact and express an array of different enzymes to degrade this recalcitrant material (de Boer et al. 2005; Burns et al. 2013; López-Mondéjar et al. 2019). Hyphal growth of fungi allows access to cellulose fibers via pores in the cell wall (de Boer et al. 2005). Hyphae bridge air filled voids in the soil and cross nutrient-poor spots if nutrients are heterogeneously distributed. The environment around the hyphae—the mycosphere—is a hotspot of microbial activity in soils (de Menezes et al. 2017). Bacteria are known to interact with fungal hyphae, using them as fungal highways to improve their motility in soils, as a substrate for biofilm formation and sometimes also as a nutrient source (Deveau et al. 2018). Furthermore, bacterial biofilms in the soil form on clay particles, roots or decomposing organic material (Burmølle et al. 2012) and soil microbes also exist self-immobilized in the form of small micro-aggregates (Cai et al. 2019). Soil biofilms play a dominant role in soil ecology and the degradation of decaying organic material (Costa et al. 2018; Burmølle et al. 2012). However, information on biofilm structure and the role and the mode of interaction of bacterial and fungal community members is scarce due to the experimental challenges in studying them. Soil microbial communities exist in locally separated small microaggregates of only a few hundred cells and display a huge heterogeneity between the microaggregates (Cai et al. 2019). Furthermore, the opacity of the soil matrix hinders microscopic observation of soil biofilms (Wu et al. 2019). Soil microbes also exist as free-living planktonic cells; however, information on the distribution and functions of each fraction is limited (Bystrianský et al. 2019).

### Lignocellulose degradation in predominately anaerobic ecosystems

Under anaerobic conditions, complexed as well as noncomplexed cellulases (see Box 1) are expressed to convert lignocellulosic substrates to a variety of final products including CO_2_, CH_4_, H_2_, and organic acids, while cell mass production is low (Wei et al. 2009). Typical anaerobic ecosystems for lignocellulose destruction are for example the rumen, aquatic sediments, landfills, or anaerobic digesters.

The complex microbiome inside the rumen of ruminant animals enables the conversion of lignocellulosic biomass such as grasses or twigs to short chain fatty acids (SCFAs) and to microbial biomass as energy and protein sources for the hosts (Brulc et al. 2009). The rumen contains some of the most cellulolytic mesophilic microbes described from any habitat (Hess et al. 2011). Around two thirds of hay for example is degraded in the digestive tracts of cows (Ineichen et al. 2019). In the rumen, the majority of the microorganisms—around 70%—are attached to the solid feed particles and live in a biofilm (Weimer et al. 2009; Mason and Stuckey 2016; Akin and Rigsby 1985). These complex communities are dominated by bacteria, but anaerobic fungi, archaea, protists, and viruses also contribute critical functions to the communities (Leng 2017). A vast majority of rumen species are not yet culturable, but culture-independent omics studies allowed to gain insight into the community composition and function (Chaucheyras-Durand and Ossa 2014). It is estimated that 7000 different bacterial species and 1500 archaea exist in rumen environments with *Firmicutes* (mainly *Clostridia*), *Bacteroidetes*, and *Proteobacteria* being the most common phyla (Brulc et al. 2009). In a single animal though, around 150 to 250 taxonomic units are found and there is a large variety in consortia composition (Brulc et al. 2009). Nevertheless, a high phylogenetic similarity between individual rumen samples was demonstrated together with a small core microbiome that was shared between individual ruminants of the same species (Jami and Mizrahi 2012). Even cross-species, a core microbiome was found, and it was shown that the diet of a ruminant had a larger effect on the community composition than the type of the host (Henderson et al. 2015).

The rumen microorganisms form complex multispecies biofilms by a sequence of events: when feed particles enter the rumen, microbes associate—randomly or as a response to a chemoattractant—with damaged surface sites created by the initial chewing of the ruminant. Microorganisms attach to nutrient niches which are favorable for them, proliferate, and form microcolonies by producing EPS. The release of different products such as sugars or H_2_ attracts secondary microorganisms that proliferate as well and establish themselves in a suitable niche of the maturing biofilm. Formation of such spatially structured consortia is very fast and occurs within the first 2 h after feed intake (Leng 2017). The biofilm mode of living increases the rates of all reactions involved in fermentation as it allows the close cooperation of microorganisms. Especially the avoidance of feedback inhibition by H_2_ on the cellulolytic bacteria by the efficient removal of H_2_ through conversion to methane by syntrophic methanogenic archaea is one critical factor for the efficient digestion of feed particles (Leng 2011; Mason and Stuckey 2016). Furthermore, rumination of partly digested feed is beneficial for efficient digestion. Through rumination, the biofilm as well as trapped CO_2_ (which causes local pH drop) is removed (Mason and Stuckey 2016) and new surfaces are exposed that are colonized by suitable consortia, which are different from the initial ones (Edwards et al. 2008).

Anaerobic gut fungi—*Neocallimastigomycota*—account for up to 8% of the microbial mass of the gut (Hooker et al. 2019; Theodorou et al. 1996) and are the primary microbes colonizing plant biomass while the other microbiota are getting involved later (Haitjema et al. 2014; Orpin 1975). Anaerobic gut fungi degrade untreated biomass through invasive growth of their rhizomycelium into and through the particles (Lillington et al. 2019) and are able to solubilize 40 to 70% of lignocellulose in 4 days in in vitro digestion experiments employing rumen fluid supplemented with antibiotics (Akin and Rigsby 1987). Some isolated strains grew on non-pretreated grasses at rates comparable or even higher to the ones on soluble substrates (Solomon et al. 2016). Anaerobic fungi encode significantly more CAZymes than *T. reesei* or *Aspergillus* species. Through horizontal gene transfer, they integrate both fungal and bacterial hydrolytic strategies and secrete extracellular catalytic complexes similar to a cellulosome (Haitjema et al. 2017). It has been suggested that the hyphae of the *Neocallimastigomycota* are closely associated with the EPS at the base of the fermentative biofilm. Hydrogen produced by the fungi is then consumed by the archaea in the biofilm (Leng 2017). This hypothesis is supported by a study that demonstrated that a monoculture of *Neocallimastix frontalis* solubilized only 16% of crystalline cellulose in 72 h, while a co-culture with *Methanobrevibacter smithii* solubilized 98% in the same time (Wood et al. 1986)

While biofilms are essential for fiber degradation in the rumen, their impact in digestive tracts of termites is less clear. Termites can rapidly mineralize lignocellulose and 74 to99% of the cellulose is removed during the passage through their guts. It is known that biofilms form on the cuticle of the hindgut, but no evidence was found in the literature that biofilms are also formed on the particles (Brune and Dietrich 2015; Brune 2014).

In anaerobic digesters the role of biofilms and the distribution of solid associated and planktonic microbial populations is less investigated than in rumen ecosystems. Several researchers verified the existence of biofilms containing, e.g., *Fibrobacter* or *Clostridia* on solid substrates in anaerobic digestion experiments (McDonald et al. 2012; O’Sullivan et al. 2005; Song et al. 2005). Jensen et al. (2008) estimated that during anerobic digestion only 25% of the microbial biomass was substrate bound, which is a much smaller fraction than observed in the rumen. The authors argued that in anaerobic digestion more soluble substrates such as SCFAs are present, as they are not absorbed during the process, which supports a larger planktonic fraction.

### Fundamental investigations on defined anaerobic cellulolytic biofilms

Due to the importance of multispecies biofilms for cellulose digestion in the biosphere and the difficulties to study this highly complex aggregate in the laboratory, several research groups explored the role and function of anerobic defined bacterial biofilms composed of one or only a few types of microorganisms. Many anaerobic, cellulolytic bacteria form biofilms on cellulosic substrates, e.g., *Clostridia* such as *C. phytofermentans* (Warnick Thomas 2002; Tolonen et al. 2011; Jain et al. 2013), *C. thermocellum* (Dumitrache et al. 2013a; Wang et al. 2011), *C. celerecrescens*, and *C. cellulolyticum* (Pantaléon et al. 2014), and non-clostridial species such as *Fibrobacter succinogenes* (Gong and Forsberg 1989) and *Ruminococcus albus* (Weimer et al. 2006; Kudo et al. 1987). The biofilm allows for a high concentration of cellulases at the boundary layer and a more complete capture of hydrolysis products directly at the hydrolysis site, which is energetically favorable (Dumitrache et al. 2013a).

**Fig. 3:**
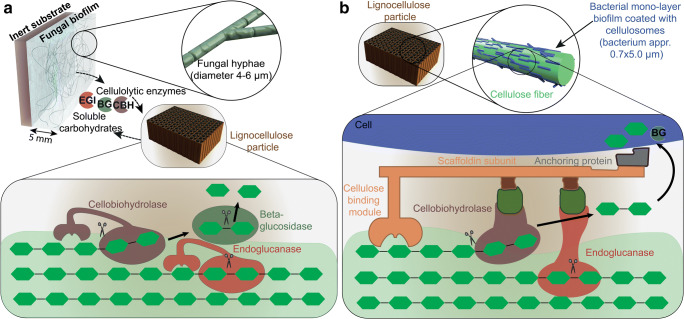
Schematic overview of the formation of fungal and bacterial biofilms and representations of the enzymatic hydrolysis of lignocellulose particles by non-complexed fungal cellulases and by cell wall bound bacterial cellulosomes. **a** Fungal hyphae can grow in the submerged state or might form a biofilm, for example on an inert substrate. Fungal biofilms can reach multiple millimeters in thickness. Fungi produce and secrete non-complexed cellulolytic enzymes. The fungal enzyme cocktail might contain endoglucanases, cellobiohydrolases and β-glucosidases which catalyze the hydrolysis of cellulose to glucose monomers. These glucose monomers diffuse to the fungal biofilm and serve as carbon source for the fungus. **b** Cellulolytic bacteria typically express free or cell-bound cellulosomes—enzyme superstructures where different catalytic subunits are linked via dockerin and cohesion domains to a scaffoldin. To enable spatial proximity to the insoluble substrate, cellulolytic bacteria form a monolayer biofilm directly on the lignocellulose particle or on the cellulose fiber, respectively

*C. thermocellum* is one of the most studied cellulolytic anaerobic bacteria and is a promising candidate for the direct conversion of lignocellulose to fuels and chemicals due to its high growth rate (0.1–0.16 h^−1^) on crystalline cellulose (Dumitrache et al. 2013a). In flow cells, where the cellulosic substrate is retained but the dilution rate is much higher than the growth rate of planktonic cells, the characteristics of substrate bound cells can be studied without any interference from planktonic cells, as these are washed out of the flow cell. It has been shown, that *C. thermocellum* biofilms alone can achieve near-complete substrate hydrolysis in such flow cells (Dumitrache et al. 2013b). Advanced non-disruptive in situ imaging revealed that the cells formed over time a confluent monolayered biofilm directly on the substrate, but without the characteristic EPS matrix (Fig. 3b). The cells were mainly oriented parallel to the cellulose fibers, but with increasing biofilm density also perpendicular relative to the axis of the cotton fiber (Dumitrache et al. 2013a). A similar cellulose colonization pattern was observed for *Fibrobacter* (Kudo et al. 1987). In contrast, *C. phytofermentans* colonized the cellulose fiber without a preferred orientation (Zuroff et al. 2014). Even in the presence of planktonic cells, cellulose degradation is synchronized with biofilm formation, e.g., only the areas of cellulose surface colonized by *Caldicellulosiruptor obsidianis* were significantly degraded (Wang et al. 2011). The surface mode of cellulose degradation has a significant influence on the rate of this process, as could be shown by measuring real time CO_2_ production profiles. For cellulose hydrolysis by *C. thermocellum*, these profiles revealed different phases: in the first phase, the cellulose is colonized with the biofilm until full coverage is reached. In this time, the hydrolysis rate is determined by the number of microbes attached on the surface and is thus increasing over time. Following is a phase with a constant hydrolysis rate that is determined by the available surface area that can be covered with a monolayer biofilm. During this phase, the biofilm cell mass is constant and cellulose sheets are reduced in thickness (Dumitrache et al. 2013b).

An analysis of the fate of the released soluble sugars revealed that depending on the carbon loading 13.7 to 29.1% of the hydrolyzed cellulose was not metabolized by the biofilm but washed out of the flow cell with the liquid stream (Dumitrache et al. 2013a). In a batch system, these sugars would be consumed by the planktonic cells. Indeed, Dumitrache et al. (2017) showed that sugar concentrations are below approximately 0.03 g L^−1^ in the liquid phase and concluded that the planktonic cells are carbon-limited. The authors also demonstrated profound differences in gene expression of sessile and planktonic *C. thermocellum* cells. Of all analyzed genes, 59.3% had an at least 2-fold different expression level. For instance, sessile cells had significantly greater expression of genes involved in carbohydrate catabolism and in critical functions for cell division, while planktonic cells overexpressed genes for flagellar motility (Dumitrache et al. 2017). Correspondingly, experimental cellulase activity measurements using a fluorescent substrate found a nearly four times higher cellulase activity per cell in surface attached cells compared to planktonic cells (Morrell-Falvey et al. 2015).

Inspired by the positive effect of rumination on feed digestibility, Balch et al. (2017) investigated the impact of continuous ball milling on the solubilization of senescent switchgrass in fermentations by *C. thermocellum.* The authors demonstrated that the total carbohydrate solubilization could be increased from 45% without ball milling to 88% by in situ ball milling.

### Cellulase production by aerobic fungal biofilms

Aerobic filamentous fungi such as *Trichoderma, Aspergillus*, *Penicillium*, or *Fusarium* are the main producers of cellulases on industrial scale. Here, submerged fermentation in stirred tank reactors on soluble substrates is the standard mode of operation due to the good control options and ease of operation (Singhania et al. 2010). Alternatively, cellulases can be produced by fungal biofilms. The existence of such biofilms has been debated (Harding et al. 2009), but it is now established, that fungi can indeed form biofilms and produce EPS (Flemming and Wingender 2010; Pesciaroli et al. 2013; Flemming and Wingender 2001). However, throughout the literature, there is no distinct definition of what and what not constitutes a fungal biofilm and often the term “biofilm” is not mentioned, even though one likely exists in the reported growth mode. In the following, we assume that biofilms are formed in every cultivation, where fungi colonize solid substrates. Thus, this definition includes biofilm fermentations, where the biofilm is formed on an inert surface and is submerged in the medium as well as solid state fermentations, where the fungus is growing on the solid feedstock or an inert surface in the absence of a free water phase.

Fungal solid-state fermentations have attracted considerable research interest as a cost-efficient valuable alternative to submerged fermentations for the production of cellulolytic enzymes (Hölker et al. 2004; Yoon et al. 2014; Singhania et al. 2010). A range of nonedible cellulosic substrates such as wheat bran, corn cobs, banana waste, or wheat straw were fermented with different fungi and bacteria, such as *T. reesei*, *A. niger*, *Bacillus subtilis*, *Penicillium decumbans*, or *Thermoascus auranticus*. Comparisons of achieved enzyme activities with those of free mycelial fermentations are seldom reported, but for example, Chahal (1985) reported a 72% higher cellulase yield (see Table 1). Overall, a 10-fold cost reduction for cellulase production in solid-state fermentation compared to submerged fermentation has been estimated (Tengerdy 1996). Recently, Zhao et al. (2019) performed transcriptomic profiling of the filamentous fungus *Penicillium oxalicum* during solid-state and submerged fermentation and demonstrated, that the expression of major cellulase genes was higher under solid state conditions, while genes involved in the citric acid cycle were downregulated.

**Table 1:** Comparison of performance of biofilm-based cellulase and β-glucosidase production with free mycelial cultivation. PDMS, polydimethylsiloxane; CDW, cell dry weight; FP, filter paper

Microorganism(s)	Target enzyme	Mode of fermentation and substrate	Enzyme activity in biofilm fermentation (difference to submerged)	Activity in submerged, free mycelial fermentation	Fermentation time (h)	Reference
*A. niger* ATCC 10864	Cellulase (FPase)	Submerged biofilm on perlite, lactose as carbon source	1786 FPU L^−1^ (+ 53%)	1165 FPU L^−1^	72	(Gamarra et al. 2010)
		Solid-state fermentation on perlite, lactose as carbon source	1174 FPU L^−1^ (+ 1%)		72	
*A. niger* ATCC 10864	Cellulase (FPase)	Submerged biofilm on polyester fabric in micro-bioreactor, lactose as carbon source	5237 FPU L^−1^ (+ 205%)	1717 FPU L^−1^	96	(Villena and Gutiérrez-Correa 2006)
*T. reesei RUT C-30* (VTT: D-86271)	Cellulase (FPase)	Submerged biofilm on PDMS membrane, Avicel	1.4 FPU mg_CDW_^−1^ (difference not significant)	1.2 FPU mg_CDW_^−1^	168	(Xiros and Studer 2017)
	β-Glucosidase		19 mU mg_CDW_^−1^ (+ 280%)	5 mU mg_CDW_^−1^	96	
*A. phoenicis* (VTT: D-76019)	β-Glucosidase		650 mU mg_CDW_^−1^ (+ 225%)	200 mU mg_CDW_^−1^	144	
*T. reesei* QMY-1	Cellulase	solid state fermentation on wheat straw	250–430 IU g^−1^_cellulose_	160 to 250 IU g^−1^_cellulose_	528	(Chahal 1985)

In the few reported data where biofilms on inert surfaces are employed for cellulase production, enzyme titers were consistently higher or at least as high as those reached in free mycelial fermentations (see Table 1). Especially, β-glucosidase activities produced by both *A. niger* and *T. reesei* were much higher in the biofilm fermentation. Besides higher enzyme titers, 3 to 4 times higher transcriptional expression of selected genes encoding for lignocellulolytic enzymes have been measured by Gutiérrez-Correa et al. (2012) for biofilm cultivations. Biofilm immobilization of filamentous fungi also allows continuous fermentations at dilution rates that are higher than the washout dilution rates of freely suspended cells. Webb et al. (1986) showed that *T. viride* formed a biofilm on stainless steel spheres in continuous cellulase production using glucose as substrate. The volumetric productivity and the yield of cellulase were 53% and 35%, respectively, higher than in the batch system with free mycelium.

### Biofilm-based consolidated bioprocessing of lignocellulose

The development of conversion processes of non-edible lignocellulosic biomass to a variety of chemicals is an important measure to enable society’s transition from a petroleum-based to a bio-based economy. One promising process configuration is consolidated bioprocessing (CBP), where all biochemical steps (the production of the cellulolytic enzymes, enzymatic hydrolysis of the polymeric carbohydrates and the fermentation of the resulting sugars to the desired product) are integrated in one reactor. CBP can be based on complexed or noncomplexed cellulolytic systems and for both cases examples for biofilm-based approaches are reported.

Biofilm forming *C. thermocellum* strains have emerged as one of the most promising CBP hosts to be engineered for the desired product forming capabilities as they belong to the most effective strains in solubilizing native or pretreated lignocellulosic biomass (Holwerda et al. 2019; Paye et al. 2016). For example, the highest reported ethanol titer achieved with a monoculture was 14 g L^−1^ applying an engineered *C. thermocellum* strain growing on 40 g L^−1^ pure microcrystalline cellulose (Argyros et al. 2011). In a co-culture together with *T. saccharolyticum*, the titer increased to 38 g L^−1^ ethanol achieved in 146-h fermentation time using 92 g L^−1^ Avicel. Higher alcohols have been produced as well by monocultures of engineered strains, but titers are much lower: for example, 0.66 g L^−1^ isobutanol were produced in 9 days by an engineered *Clostridium cellulolyticum* (Higashide et al. 2011) or 0.38 g L^−1^ n-butanol produced within 120 h by *C. thermocellum* (Tian et al. 2019). As an alternative to the construction of a single CBP strain, consortia consisting of cellulolytic and product forming specialists have been successfully engineered. An anaerobic co-culture of the cellulolytic strain *Clostridium cellulovorans* and the non-cellulolytic, solventogenic bacterium *Clostridium beijerinckii* could produce 12 g L^−1^ butanol, ethanol, and acetone (ABE) from pretreated corn cobs in 80 h (Wen et al. 2014). After targeted further genetic optimization of both consortium members, the titer could be increased to 22.1 g L^−1^ ABE solvents reached in 109 h in a fed-batch fermentation of pretreated corn cobs (Wen et al. 2017). Butyric acid could be produced by combining *C. thermocellum* with the thermophilic butyric acid producing *C. thermobutyricum*, achieving a yield of 33.9 g L^−1^ in 25 days using delignified rice straw at a temperature of 55 °C (Chi et al. 2018).

In our group, we developed a consortium based CBP concept that utilizes aerobic cellulase production by a *T. reesei* biofilm in a membrane aerated reactor and different anaerobic fermenting microorganisms. Oxygen necessary for the growth of *T. reesei* is fed through a polydimethylsiloxane membrane, which also serves as the inert surface for biofilm formation (Fig. 3a). The metabolic activity causes an oxygen gradient within the biofilm and leads to anaerobic conditions in the upper part of the biofilm as well as in the liquid bulk phase. The general feasibility of the concept was successfully demonstrated by producing 9.8 g L^−1^ ethanol in 144 h (67% yield) from pretreated wheat straw using the glucose fermenting *Saccharomyces cerevisiae* and the xylose metabolizing *Scheffersomyces stipites* (Brethauer and Studer 2014). If the facultative anaerobe *Lactobacillus pentosus* was employed as the fermenting strain, up to 19.8 g L^−1^ lactic acid from nondetoxified pretreated beech wood and up to 34.7 g L^−1^ lactic acid from 50 g L^−1^ microcrystalline cellulose could be produced in 200 or 215 h, respectively (Shahab et al. 2018). In order to produce mixed short-chain fatty acids (SCFAs), a natural rumen microbiome was employed instead of defined fermenting microorganisms. At 30 °C, the presence of a *T. reesei* biofilm increased the acid concentration by 39% (7.3 g L^−1^ SCFAs produced in 360 h) compared to the case with the rumen microbiome alone (5.1 g L^−1^ SCFAs) using 15 g L^−1^ pure crystalline cellulose. The beneficial effect of the fungal biofilm on the process yields and productivities was attributed to the enhanced cellulolytic activities compared with those achieved by the rumen microbiome alone (Xiros et al. 2019).

## Conclusion

Taken together, microbial biofilms have a considerable impact on lignocellulose degradation. Anaerobic bacteria typically form without the synthesis of EPS very thin, often monolayered biofilms on the cellulose surface, which are essential for efficient cellulose solubilization. Such cellulolytic biofilms allow for a high concentration of enzymes at the boundary layer between the solid substrate and the liquid phase and the fast capture of hydrolysis products directly at the hydrolysis site. In contrast, aerobic fungal biofilms typically form much thicker biofilms that act as external digestion systems by immobilizing non-complexed enzymes, solid substrates and soluble hydrolysis products in the EPS matrix. For both systems, enhanced expression of genes for carbohydrate active enzymes as a response to the attachment on solid substrate has been demonstrated.

In the biosphere, efficient aerobic and anaerobic degradation systems have evolved to overcome the recalcitrance of lignocellulose towards microbial degradation. It has been shown that complex multispecies biofilms play a crucial role in the deconstruction of lignocellulose, but we have only just begun to understand the complex interactions between the multitude of microorganisms from different kingdoms that enable such efficiency. A thorough understanding of these complex systems might enable the transfer of important paradigms in order to improve engineered bioprocesses. Successful examples for this include, e.g., the addition of H_2_ consuming microorganism to an anaerobic fungus or the in situ milling of biofilm colonized substrates. The targeted application of biofilm systems for lignocellulose conversion processes is still underexplored but is a promising route especially regarding the engineering of artificial microbial communities as biofilms facilitate beneficial microbial interactions and allow for the creation of a suitable ecological niche for each member (Shahab et al. 2020).

Box 1 Enzymatic systems for cellulose degradation

In order to allow the deconstruction of recalcitrant lignocellulosic biomass, a variety of enzymes and strategies have evolved in nature, mainly based on hydrolytic glycoside hydrolases (GHs). These enzymes are classified in a system of carbohydrate active enzymes (CAZy) based on their sequence and the analysis of their structure (Lombard et al. 2014). Three different types of GHs that act complementarily and synergistically have been identified that are crucial for the degradation of lignocellulose: exoglucanases, endoglucanases, and cellobiases. While exoglucanases hydrolyze the cellulose chain from both the reducing and the non-reducing end, endoglucanases cleave glucosidic bonds within the polysaccharide chain. Cellobiases such as β-glucosidases hydrolyze the released cellobiose into two glucose monomers (see also Fig. 1). Often, the catalytic unit is connected via linker peptides to a carbohydrate binding module (CBM) (Payne et al. 2015), which enables substrate recognition at the solid liquid interface and reduces the proximity between the catalytic domain and the substrate. Aerobic fungi typically secrete free, non-complexed mono- or multifunctional cellulases, that contain one or several catalytic units (Bomble et al. 2017). In contrast, anaerobic bacteria and fungi predominately express free or cell-bound complexed cellulases, where a large number of catalytic units and CMBs are bound to a scaffold backbone and form a cellulosome. Substrate channeling in cellulosomes has been shown to enhance the cellulolytic activity over free enzymes by a factor of 12 (Lillington et al. 2019).

Box 2 Biofilms

Flemming and Wingender (2010) defined biofilms as “microbial aggregates that usually accumulate at solid-liquid interfaces and that are encased in a matrix of highly hydrated extracellular polymeric substances (EPS)”. EPS are natural polymers of high molecular weight primarily composed of polysaccharides, proteins, lipids and extracellular DNA (Hall-Stoodley et al. 2004). EPS are produced by a variety of microbial cells across all kingdoms including bacteria, fungi and microalgae. The chemical structure of EPS depends strongly on the producing microorganism and differs in the type of building block, chemical bonds and substituents (Leigh and Coplin 1992). Generally, biofilm producers partition around 20% of the substrate carbon into EPS production (Kroukamp and Wolfaardt 2009). EPS stabilize the structure of biofilms and form the scaffold for the three-dimensional architecture (Flemming and Wingender 2001). The macroscopic appearance of biofilms varies from wet to slimy to fluffy (Flemming and Wingender 2010). Biofilms are involved in a variety of biological processes such as the initial binding of cells to solid surfaces, the formation of stable multicellular arrangements and the retention of exoenzymes and cell debris (Flemming and Wingender 2001; Czaczyk and Myszka 2007). Biofilms are the prevailing lifestyle in nature that leads to clearly distinct properties than that of planktonic cells which is also reflected by different gene expression profiles (Neumann et al. 2018; Dumitrache et al. 2017). Natural biofilms consist of highly heterogeneous multispecies consortia and allow for the self-creation of a microenvironment characterized by the presence of various physicochemical gradients. Biofilms are characterized by high cell densities and they foster intensive cell-cell communication and social cooperation. Microorganisms growing in biofilms are often more resistant to toxic compounds and biological attacks compared to planktonic cells (Flemming and Wingender 2001).
